# Occurrence of Antibiotic-Resistant Bacteria and Genes in Two Drinking Water Treatment and Distribution Systems in the North-West Province of South Africa

**DOI:** 10.3390/antibiotics9110745

**Published:** 2020-10-28

**Authors:** Collins Njie Ateba, Naledi Mahalia Tabi, Justine Fri, Marie Ebob Agbortabot Bissong, Cornelius Carlos Bezuidenhout

**Affiliations:** 1 Food Security and Safety Niche Area, Faculty of Natural and Agricultural Sciences, North-West University, Private Bag X2046, Mmabatho 2735, South Africa; frijustine2000@gmail.com; 2 Antimicrobial Resistance and Phage Biocontrol Research Group (AREPHABREG), Department of Microbiology, Faculty of Natural and Agricultural Sciences, North-West University, Private Bag X2046, Mmabatho 2735, South Africa; naledimtabi@gmail.com (N.M.T.); mabissong@yahoo.com (M.E.A.B.); 3 Department of Biomedical Science, University of Bamenda, Bamenda P.O. Box 39, Cameroon; 4 Unit for Environmental Sciences and Management, North-West University, Potchefstroom, Private Bag X6001, Potchefstroom 2520, South Africa; Carlos.Bezuidenhout@nwu.ac.za

**Keywords:** antibiotic resistance, antibiotic resistance genes, heterotrophic bacteria, potable water

## Abstract

With the increasing spread of antimicrobial resistance, there is growing attention to the contribution made by drinking water systems. The potential health impact of two drinking water treatment and distribution systems (A and B) in the North-West Province of South Africa was determined by investigating the water quality and occurrence of antimicrobial-resistant heterotrophic bacteria and genes in the raw and treated water over four seasons. Most of the physicochemical parameters except for electrical conductivity were within permissible limits. Coliform bacteria reduced from raw to potable water except for counts higher than the threshold recorded in Summer and Winter. A total of 203 heterotrophic bacterial isolates were recovered on chromogenic R2A medium and subjected to susceptibility testing to twelve antibiotics. Most of the isolates were resistant to β-lactam antibiotics and Trimethoprim, whereas they were susceptible to Ciprofloxacin, Erythromycin, and Neomycin. The proportions of Cephalothin and Kanamycin-resistant isolates were significantly higher (*p* < 0.05) after treatment for site A, compared to significantly lower β-lactam, Oxytetracycline, and Trimethoprim-resistant isolates for B. Over 50% of isolates were of high risk, indicating their origin from high antibiotic-use sources. Seventy-one (35%) isolates were multidrug-resistant, out of which the majority (53.5%, *n* = 38) possessed the *strA* gene, followed by *strB* 21 (29.6%), *dfrB* 13 (18.3%), *aadA* 11 (15.5%), *bla*_CTX-M_ 5 (7.0%), and *tetA* 3 (4.2%). The 16S rRNA gene sequences of the isolates revealed strains belonging to eight bacterial families, some of which are clinically important.

## 1. Introduction

Water is essential to all living organisms and is a fundamental human right. An adequate supply of potable water remains a great socioeconomic problem in most developing countries [1]. South Africa is situated in a semi-arid region and is classified as a water-stressed country with very few surface waters. Due to this challenge, some municipal regions employ the water re-use strategy, which involves the use of treated wastewater to provide potable water to consumers. Wastewater is generated from both industrial and domestic activities and therefore contains a variety of compounds including gasoline additives, surfactants, endocrine disruptors, pharmaceuticals, and personal care products [2,3].

The frequent and unregulated use of antibiotics in human and veterinary medicine plays a key role in the incessant increase in antimicrobial resistance globally. A direct consequence of the unregulated use is the increased detection of antimicrobial metabolites in the environment, which imposes a selective pressure on environmental bacteria, enhancing the development of antibiotic-resistant bacteria (ARB) and genes [4,5]. Human, animal and environmental components (One Health) interact and contribute directly or indirectly to the rapid emergence and spread of antimicrobial resistance in surface and subsurface waters. However, hotspot sources where bacteria regularly encounter various antibiotic doses include livestock farming, aquaculture, hospitals, and other health care facilities and wastewater treatment plants (WWTPs) [6,7,8,9]. Untreated sewage leakages have also been recognized as a source of antibiotic resistance genes (ARGs) and ARB to the environment, most of which end up in environmental waters. Although difficult to estimate, it is worth mentioning that hotspot sources such as livestock farming and aquaculture are reported to use much higher amounts of antibiotics than the total amount used in medical settings [9,10,11]. In addition, a hot spot such as WWTPs receives water from a variety of sources including hospital wastewater, obviously containing among other contaminants, pharmaceuticals. ARGs and ARB from WWTPs can reach aquatic environments such as rivers, dams, and other subsurface waters through the discharged final effluents [4,12]. Through horizontal gene transfer (HGT), antibiotic-resistant environmental bacteria easily transfer their resistance to human pathogens. Unfortunately, both wastewater and drinking water treatment processes do not totally eliminate ARB and ARGs. Activities such as bathing, swimming, the consumption of irrigated food produce, or exposure to irrigation water from these water sources constitute risk exposure sources to humans. These pathogens pose significant public health threats worldwide, unlike their drug-sensitive counterparts, leading to prolonged hospital stay, elevated health cost, and increased mortality [13].

In the North-West Province of South Africa, with high industrial activities, particularly mining and farming, there are challenges resulting in low water availability and deteriorating water quality [14]. Furthermore, technical difficulties resulting from poorly maintained water treatment infrastructures as well as service delivery backlogs may compromise the production of adequate potable water. Following purification, mechanical failure in distribution systems might also expose the water to various conditions that may alter the quality status [15]. In addition, of concern is the development of biofilms that may result in an ineffective disinfection of pipe material, microbial resistance to disinfectants, and breakage in pipelines [16,17]. Generally, potable water should not pose any significant health risk over a lifetime for which it is consumed and should not also produce any hypersensitive reactions despite the immune status of the consumer. Therefore, following World Health Organisation (WHO) recommendations, a preventative risk management approach is adopted whereby the entire water supply system from source to tap is evaluated in efforts to identify possible risks as well as engaging in risk management.

In the past, only pathogens associated with diseases such as cholera and typhoid fever were of great concern in water contamination. More recently, especially in the last two decades, newly recognized microbial contaminants pose more significant challenges to water safety and strains harboring antibiotic resistance determinants and virulence genes earmarked as severe public health risk [18,19]. The overwhelming spread of antibiotic resistance has been attributed to its frequent use and misuse in human medicine, agriculture, and aquaculture, resulting in the accumulation of metabolites in various ecosystems, including environmental waters [20,21]. Although bacterial genetic alterations result in the development of antimicrobial resistance, the acquisition of antibiotic resistance factors through horizontal gene transfer (HGT) is the frequent means of its spread to previously susceptible bacteria. Various studies conducted in the North-West Province of South Africa revealed pathogenic, opportunistic, and antibiotic-resistant bacterial species in potable water as well as in unprotected water catchments that are used for drinking and other household activities [22,23,24]. However, these studies focused on species-specific cultures, and data generated from such studies are likely to be biased due to failure to consider the total bacterial population. It is worth mentioning that other heterotrophic bacteria species harboring a wide range of antibiotic resistance determinants have been recognized as water contaminants [25]. *Citrobacter* spp., *Flavobacterium* spp., *Acinetobacter* spp., *Micrococcus* spp., and *E. coli* as well as opportunistic pathogens belonging to the genus *Aeromonas*, *Klebsiella*, and *Pseudomonas* have been implicated as frequent contaminants in water sources [26]. Therefore, this study was aimed primarily to determine the occurrence of antibiotic-resistant heterotrophic plate count (HPC) bacteria and their associated antibiotic resistance genes (ARGs) in two water distribution systems in South Africa.

Although the use of chlorination is of benefit for the production of safe drinking water, orthodox water purification techniques may not always be effective at removing bacterial cells [2,27,28,29]. Chlorination may also promote the increase of ARGs and free bacterial DNA in the environment. Antibiotic-resistant bacteria (ARB) have been reported to be more resistant to chlorination [30,31]. Therefore, this adds to the need to continually monitor the occurrence of ARB in water treatment facilities and their distribution systems.

The provision of quality water for personal hygiene, consumption, and use in medical settings is paramount. Water quality describes the physicochemical, biological, and aesthetic properties that define its suitability for a variety of uses and for protecting consumer health [19]. Although some water contaminants can be identified using its aesthetic properties, most others can only be determined using other water quality measures such as physicochemical and biological parameters. Safe drinking water should adhere to recommended standards that pose no major concerns on public health [15,19]. The presence of microbiological indicators in drinking water such as fecal coliform and the thermotolerant coliform bacteria (*E. coli*) show evidence of contamination and the possible presence of pathogenic inhabitants, especially those that can cause gastrointestinal diseases [15,19]. An estimated 88% of deaths worldwide are due to water-related diseases such as diarrhea, with approximately 90% of deaths recorded in children under the age of five [32]. Other infections of the skin, eye, ear, and upper respiratory tract ensue through the exposure of skin abrasions and abscesses to contaminated water [33]. Thus, poor water quality can pose health risks to users. Therefore, this study also assessed the water quality in the distribution systems using physicochemical and microbiological indicators.

## 2. Results

### 2.1. Physicochemical Water Quality Parameters

Water temperatures were higher during the warmer than the cooler seasons, as expected, and they were also higher at the distributions than the raw water samples. All pH values were above neutral, and a majority were within stipulated limits (≥5 to ≤9.7) except for the raw (pH 9.94) and inlet water (pH 9.93) readings during Spring at site A, which were slightly higher. The electrical conductivity (EC) values ranged from 239 to 846 µS/cm and 262 to 805 µS/cm for sites A and B, respectively, exceeding recommended limits [34]. The lower values were obtained in Summer as opposed to higher Winter readings. Water collected from D2, which was intended for direct human consumption, had the highest reading of 876 µS/cm. The total dissolved solids (TDS) values were highest in Autumn (717–816 mg/L) compared to Summer (152.3–183.2 mg/L). However, throughout the sampling period, all TDS readings were below the threshold value of ≤1200 mg/L. The results obtained for the mean readings of the physicochemical water quality parameters for the different sampling sites and seasons are shown in Table 1.

### 2.2. Bacteriological Water Quality

The means of the bacteriological water quality parameters of the treated water samples were compared to the quality guidelines limits for drinking water [34]. Generally, total coliform (TC), fecal coliform (FC), and *E. coli* count in the raw water samples at site A ranged from 1 to 17, 2 to 17, and 2 to 24 cfu/100 mL, respectively, while their corresponding counts at site B ranged from 1 to 52, 7 to 12, and 1 to 62 cfu/100 mL. These bacteria coliforms were prominent in untreated water samples and reduced to a few or undetectable levels for the consumer samples as expected. An exception was the consumer sample D1, at site B with TC counts during Winter and Summer of 21 cfu/100 mL and FC counts of 9 cfu/100 mL (Table 2). These values are above the stipulated requirement of no fecal coliform or <10 cfu/100 mL TC in water considered safe for drinking [34]. Although lower than the former, a similar trend for fecal coliform counts was also observed for the consumer tap sample D1 at site A for these two months. Remarkably, no *E. coli* was detected in the consumers’ samples throughout the sampling period. The outcome of the mean microbial water parameters for the various sampling sites and seasons is shown in Table 2.

### 2.3. Antimicrobial Resistance Profiles of Heterotrophic Bacterial Isolates

Heterotrophic bacteria were isolated from all water samples. A total of 203 isolates, which included 102 isolates (33 Gram-negative and 69 Gram-positive) recovered from site A and 101 (31 Gram-negative and 70 Gram-positive) from site B were selected for antimicrobial resistance screening. The antimicrobial resistance patterns were analyzed as a function of the site (A and B) and the condition of the water (treated and untreated). Thirty-nine of the 40 water samples contained at least an antimicrobial-resistant (AMR) isolate.

Generally, a large proportion of the isolates were resistant to β-lactam antibiotics (Ampicillin and Penicillin G). While the proportion of resistant isolates from site B were higher “before treatment” compared to “after treatment” for most of the antibiotics tested (9/12), the reverse was true for site A. For example, at site A, a prevalence of 53.6% and 42.9% resistance to Ampicillin and Penicillin G respectively “before treatment” increased to 61% and 58.5% “after treatment”. On the other hand, at site B, an appreciable proportion of isolates resistant to Ampicillin (92.6%) and Penicillin G (70.4%) “before treatment” dropped to 55.8% and 39.5% respectively “after treatment”. A considerable percentage (42.5–67.5%) of resistance to Trimethoprim was also recorded from both sampling sites with a resulting overall resistance of 51.7% (105/203). Significantly higher (*p* < 0.05) proportions of Cephalothin (*p* = 0.0) and Kanamycin (*p* = 0.035) resistant isolates were recorded after treatment compared to the raw water isolates at site A, while at site B, lower proportions of Ampicillin (*p* = 0.001), Penicillin G (*p* = 0.012), Oxytetracycline (*p* = 0.01), and Trimethoprim (*p* = 0.011) were noted. Ciprofloxacin was the most effective antibiotic against the isolates, with only one isolate in each category presenting resistance traits to the drug. This was closely followed by resistance to erythromycin (3.9%, *n* = 8), cephalothin (5.9%, *n* = 12), and neomycin (7.4%, *n* = 15). Table 3 shows the proportion of antibiotic-resistant isolates obtained from the drinking water treatment and distribution systems.

A total of 71 (35%) isolates were multidrug-resistant (MDR). These constituted 30 isolates from site A and 41 from site B. At site A, resistance to 3, 4, 5, 6, 7, and 8 antibiotics were recorded in 4, 10, 10, 4, 1, and 1 isolate(s), respectively, while at site B, these multidrug-resistant traits occurred in 5, 12, 12, 9, and 3 isolates, respectively. The most frequent MDR phenotype was AP^R^-PG^R^-CHL^R^-TMP^R^, which was observed in 4 and 3 isolates from sites A and B, respectively. MARI values > 0.2 were recorded by a total of 102 isolates including 45 (44.1%; 45/102) from site A and 57 (56.4%) from site B, indicating isolates from high-risk sources; i.e., the isolates originated from areas of high antibiotic use.

### 2.4. Extracellular Enzyme Production

Pathogenic bacteria produce enzymes to carry out their pathogenic effects in their host. The potential of the MDR isolates to produce hemolysin was revealed by a total of 53 (74.6%) isolates, and the majority were β-hemolytic (52.1%, *n* = 37), 16 (22.5%) were α-hemolytic, and the rest (21.1%, *n* = 15) displayed no hemolytic patterns on blood agar (γ-hemolytic). On the other hand, 50 (70.4%) of the isolates were lipase positive, 35 (49.3%) showed protein catabolism activity, while only three (4.2%) isolates were able to degrade nucleic acids.

### 2.5. Prevalence of Antibiotic Resistance Genes in MDR Isolates

The MDR isolates were subjected to specific PCR assays designed to screen for the presence of 13 antibiotic resistance determinants, out of which six were positively amplified. Most of the isolates (78.9%, *n* = 56) possessed at least one of the genes tested. The bulk of them carried the *strA* gene fragments (53.5%, *n* = 38) followed by *strB* (29.6%, *n* = 21), both coding for resistance to Streptomycin. On the other hand, the genes that encode resistance to Trimethoprim (*dfrB1*, *dfrB2*), aminoglycosides (*aadA*), beta-lactamase (*bla*_CTX-M_), and Tetracycline (*tetA*) were detected in 13 (18.3%) 11(15.5%), 5 (7%), and 3 (4.2%) isolates, respectively. An isolate MDW1 from the raw water sample of site A harbored up to five resistance genes: *strB*, *aadA*, *tetA*, *bla*_CTX-M_, and *dfrB*. Representative agarose gels showing some of the PCR amplified fragments of the antibiotic resistance genes detected are shown in Figures S1–S4 (Supplementary Materials).

### 2.6. Identification of MDR Isolates Based on 16S rRNA Gene Sequences

Bacterial 16S rRNA sequences of 33 representative MDR heterotrophic bacterial isolates revealed 29 isolates that possessed 92–100% similarities to previously characterized species belonging to the bacterial families: Enterobacteriaceae, Xanthomonadaceae, Yersiniaceae, Bacillaceae, Pseudomonodaceae, Paenibacillaceae, Sphingomonadaceae, and Comamonadaceae. Genera/species identified included *Citrobacter* spp., *Enterobacter* sp., *Escherichia coli*, *Klebsiella pneumoniae*, *K*. *variicola*, and other *Klebsiella* spp., *Pseudoxanthomonas* sp., *Stenotrophomonas maltophilia*, Stenotrophomonas sp., *Serratia marcescens* subsp. *marcescens*, and other *Serratia* spp., *Bacillus wiedmannii* and other *Bacillus* spp., *Pseudomonas* spp., *Paenibacillus* sp., *Blastomonas* sp. and *Delftia* sp.

## 3. Discussion

The physicochemical and biological properties of water greatly influence bacterial re-growth and persistence, as well as its aesthetic and operational properties, which is why adherence to specified guidelines is important. Most of the physicochemical parameter values were within the recommended Blue Drop/WHO limits. However, of concern were the mean electrical conductivity values that exceeded the permissible limits of ≤170 µS/cm for all water samples. Electrical conductivity (EC) is the ability of a medium to carry an electric current that is reliant on the presence and concentration of dissolved solids. Therefore, enhanced by an increased concentration of ions, the relatively high electrical conductivity readings might have been a reflection of increased minerals in water [35]. High EC affects the aesthetic properties of water, especially the taste, making it unfit for drinking.

Generally, the number of bacteriological indicator organisms in the current study reduced from raw (catchment) to treated water samples. Water was of superior quality during Autumn and Spring compared to Summer and Winter. Although no *E. coli* were enumerated following purification, revealing effective purification measures, the total and fecal coliform counts in the treated and potable water from both plants during the Winter and Summer seasons violated the standards recommended for human consumption. The presence of coliform bacteria in potable water is attributed to fecal pollution and indicates the presence of pathogenic inhabitant, especially those that can cause gastrointestinal diseases such as diarrhea [15,19]. It is worthwhile to note that the catchment (dam) at site A also receives treated water from a wastewater treatment plant, while the untreated water at site B is a surface water source, around which there are some informal settlements. Therefore, these water sources may contain contaminants resulting from agricultural and anthropogenic activities noticeable only in summer and winter, which is probably due to incoming runoffs during rainfalls. The resulting contaminating bacteria must have survived treatment processes. The USEPA highlighted that some organisms survive current treatment processes, thus rendering the finished product unsafe for consumers [16]. Such water presents the risk to vehicle and spread water-related diseases.

The environment plays an important role in the development and spread of antimicrobial-resistant (AMR) bacteria and water used for recreational, agricultural, drinking, and other domestic purposes are risk exposure sources to humans [36]. Therefore, the frequent assessment of the antimicrobial resistance patterns in potable water and their sources is important for monitoring the spread, which can assist in the development of preventive strategies. In the current study, antibiotic-resistant heterotrophic bacteria were present in 98% (39/40) of the water samples screened, and 35% of the recovered isolates were multidrug-resistant (MDR). Previous studies have also reported the occurrence of antibiotic-resistant and MDR bacteria in drinking water sources [24,37,38]. The overuse and misuse, coupled with the usage of similar antibiotics in human medicine and agriculture, have been highlighted as contributing factors to the seemingly inevitable spread of antimicrobial resistance over the years [20,21]. Antimicrobial-resistant bacteria, especially MDR bacteria, complicate therapy, should infection ensue in susceptible individuals. The situation could be worsened in a country such as South Africa, where the proportion of individuals living with HIV/AIDS is high [39].

Differing from other studies, the current study also determined the antibiotic resistance prevalence in the water samples collected before and after treatment. The marked increase in the proportion of resistant isolates to Cephalothin and Kanamycin “after treatment” compared to “before treatment” is significant (*p* < 0.05), which is contrary to the expected. However, this scenario could be due to the effect of chlorine on the survival of ARB at the site supported by studies that reported higher tolerance of drug-resistant bacterial phenotypes to chlorine compared to their sensitive counterparts [27,28,29] or no substantial reduction in the ARB population [40]. Moreover, the disinfectant may directly induce the development of antibiotic-resistant traits in previously susceptible strains. An earlier report of the metagenomic analysis of chlorinated water revealed an increase in ARGs as well as other mobile genetic elements that are important in horizontal gene transfer [31]. The high total and site-specific prevalence of resistance to beta-lactam antibiotics are similar to resistances obtained for drinking water isolates in other studies [24,41]. The over 50% phenotypic resistance to the sulfonamide Trimethoprim in the current study was much higher than the 25.6% [42] and 10.9% [43] recorded previously; however, it was lower than the resistance recorded by Mulamattathil et al. [24]. Previous studies designed to evaluate the occurrence of metabolites to Trimethoprim in wastewater treatment plants revealed that there was very little elimination of this antibiotic in water samples collected during the different stages of purification [44,45], which could account for the observed prevalence. Approximately over half of the isolates in the study were from high-risk sources where antibiotics are commonly used, i.e., they originate from areas of previous exposure to antimicrobial agents. Anthropogenic activities leading to the release of antibiotic residues to the environment might have played a role [20,21,37,46]. This has a negative effect on public health especially as therapy of infections in susceptible individuals may prove infective, therefore increasing the antimicrobial resistance burden. Such strains also form a consortium of resistant traits that could be transferred to previously susceptible bacteria.

A significant proportion (53.5%) of the MDR isolates harbored the *strA* gene followed by 29.6% *strB* positive isolates. Gebreyes and Altier [47] observed a much lower percentage (30.8%) for the *strA* gene in planktonic bacteria. Streptomycin is important in the treatment of urinary tract bacterial infections caused by *E. coli* and other Gram-negative bacteria. It has been reported as an essential drug for the synergistic treatment of severe and life-threatening infections caused by *Enterococcus* species. The development of Streptomycin resistance initially documented in 1945 [48] is mediated by chromosomal mutations or the expression of streptomycin-inactivating enzymes, with the latter accounting for a large proportion of treatment failures among infections caused by clinically relevant bacteria. Both *strA* and *strB* have also been linked to the resistance of isolates to sulfonamides, although none of the isolates in the current study harbored the *sul1* gene. In addition, *strA* and *strB* have been identified in a variety of broad host range of non-conjugative plasmids within bacteria species isolated from animals and on the *Tn*3-type *Tn*5393 transposon in conjugative plasmids [46]. Generally, the presence of these resistance determinants on mobile genetic elements facilitates their transmission to susceptible bacteria. Thus, some of the ABR isolates investigated in the current study may serve as potential sources for the transmission of resistant determinants to previously susceptible bacteria species in the area.

The *aadA* gene, which also encodes resistance to streptomycin together with spectinomycin via an adenylyltransferase enzyme were present in 15.5% of the heterotrophic bacterial isolates. The presence of both *aadA* and *strA* genes were detected in three isolates. Even though two of these isolates did not possess extracellular enzymatic traits that may initiate disease in their hosts, one of the three identified as a *Bacillus wiedmannii* isolated from the water distribution system after purification was a cause for concern. The strain also possessed the *aadA* gene as well as exhibited the potential to produce the enzymes Proteinase, Lipase, and β-hemolysin. *B. wiedmannii* is highly associated with the potential to produce potent toxins and has been reported as a human pathogen [49]. Although pasteurization is capable of eliminating the pathogens in food products, especially milk, the spores are known to survive refrigeration temperatures and are capable of producing cellular pores in their host, resulting in diarrhea.

The antibiotic Trimethoprim is commonly used in the treatment of urinary tract infections, and resistance to this drug has been reported among several bacterial species, thus increasing the global burden of antibiotic resistance in humans. Trimethoprim resistance genes were detected in 18.3% of the isolates in the present study. This finding does not agree with those of a previous report in which large proportions (96% and 69%) of *E. coli* and *K. pneumoniae* harboured 13 of the *dfr-*genes analyzed [50].

Tetracycline antibiotics are most often used as broad-spectrum drugs against a wide range of bacteria, which is the reason why large proportions of isolates are usually resistant to the antibiotic. The Tetracycline resistance gene *tetA* was detected in a small proportion (4.2%) of the heterotrophic isolates compared to the 19.7% observed phenotypically to Oxytetracycline, which is one of the analogues of the Tetracycline family. This result is promising, as there has been a drop in the prevalence of resistance to the antibiotic in the study area over the years; a few years back, marked antibiotic resistances (over 70%) were observed for tetracycline amongst *E. coli* isolates from wastewater, dam, and tap water samples in the study area [37]. This is reassuring and points to a need to always consider obtaining current antibiotic resistance data to guide therapeutic choices that will have an overall positive effect on public health. Up-to-date antibiotic resistance data also assist in directing management strategies developed to prevent the spread of antibiotic resistance. The frequency of detection of extended-spectrum beta-lactamase (ESBL) encoding gene *bla*_CTX-M_ was also low. Although other ESBL-encoding genes were not considered in the current study, the low percentage in the tested gene is contrary to the increased report in these genes, especially in extended spectrum beta-lactamase-producing *Enterobacteriaceae* [51].

As supported by the ability to produce extracellular enzymes, the 16S rDNA sequences of the isolates revealed some potential opportunistic human bacterial pathogens. Examples are *Klebsiella pneumoniae* and *E. coli* belonging to the Enterobacteriaceae family. Opportunistic bacterial pathogens have been associated with nosocomial infections in clinical settings and within households. *Klebsiella pneumoniae subsp. ozaenae* has been implicated as the main causative agent of rhinopharynx chronic inflammatory disease as well as tracheo bronchopathia [52]. These isolates may pose public health complications in humans, especially in immune-suppressed persons. Another *Klebsiella* species isolated in this study, *K. variicola,* accounts for 10% of clinical *Klebsiella* infections [53]. In addition, Maatallah et al. [54], reported that *K. variicola* more so than *K. pneumoniae* was associated with higher mortality rates in humans suffering from bloodstream infections. Thus, the detection of this bacterial strain in potable water utilized by rural community residents is a cause for concern.

## 4. Materials and Methods

### 4.1. Ethical Considerations

Permission to conduct the study was obtained from the FNAS Ethics Committee of the North-West University, South Africa. Administrative authorizations and approval for sample collection were obtained from managers of the local municipality and the water treatment plants. The names of sampling sites were coded to ensure confidentiality.

### 4.2. Study Area and Selection of Sampling Points

Samples for the study included raw (untreated) and treated (potable) water samples from two major drinking water treatment and distribution systems considered as A and B in the North-West Province of South Africa. A total of forty water samples from five sampling points per site per season were collected. Samples were collected in the Winter and Spring of 2016 as well as in Summer and Autumn of 2017. For site A, sampling points included a dam (A-raw) from which water is abstracted to a water treatment plant, a sampling point at the inlet to the treatment (A-inlet), a point at outlet following treatment (A-outlet), and two randomly selected distributions (tap water) AD1 and AD2 supplied to households sourced from the treatment plant. Samples from site B were collected in a similar manner per season and therefore also consisted of the raw (B-raw), B-inlet, B-outlet, BD1, and BD2. The dam (A-raw) also receives treated water from a wastewater treatment plant, while B-raw is a reservoir from a surface water source. The treatment processes used in the drinking water treatment plant at site A include coagulation, flocculation, sedimentation, sand filtration, and chlorination, while the water at site B undergoes only sand filtration and chlorination. Potable water from both plants is used for drinking and other domestic, agricultural, and industrial purposes. Samples were collected in sterile 500 mL bottles and transported at 4 °C to the laboratory for analysis within 3–6 h of collection.

### 4.3. Determination of the Physicochemical Parameters of Water Samples

Triplicate measurements for the temperature, pH, turbidity, and electrical conductivity of the water samples were taken on site using a multi-probe digital meter (CRISON, model SensION+ MM40+, Barcelona, Spain). The mean values were compared to acceptable standards.

### 4.4. Enumeration of Indicator Organisms and Isolation of Heterotrophic Bacteria

Aliquots of 100 mL from each water sample were aseptically filtered through 0.45 µm sterile membrane filters (Pall Corporation, Port Washington, New York, NY, USA) and inoculated on mFC agar (Biolab, Modderfontein, South Africa) for the selective enumeration of fecal coliforms and Membrane Lactose Glucuronide agar (MLGA) (pH= 7.2 ± 0.2) (Oxoid Ltd., Hampshire, UK) to assay for *Escherichia coli* [55]. Plates were incubated aerobically at 37 °C for 24 h. Blue and green colonies on mFC and MLGA respectively were counted and recorded. For the isolation of heterotrophic bacteria, filters were inoculated on R2A media (pH = 7.2 ± 0.2) (Lab M, Lancashire, UK), and plates were incubated aerobically at 35 °C for up to 3 days. Isolates displaying different colonial morphologies were picked in triplicate and purified on R2A media. Pure isolates were characterized by Gram staining.

### 4.5. Phenotypic Antibiotic Resistance Testing

Twelve antimicrobial agents belonging to eight classes were used for susceptibility testing for all Gram-positive isolates. These included Ampicillin (10 µg), Penicillin G (10 U), Vancomycin (30 µg), Erythromycin (15 µg), Chloramphenicol (30 µg), Ciprofloxacin (30 µg), Cephalothin (30 µg), Streptomycin (10 µg), Kanamycin (30 µg), Neomycin (30 µg), Trimethoprim (5 µg), and Oxytetracycline (30 µg). For Gram-negative isolates, Ampicillin, Penicillin G, and Vancomycin were not tested due to possible intrinsic resistance [56]. The selection of antibiotic panel was based on their frequent usage in veterinary and human medicine. The Kirby–Bauer disc diffusion technique was used, and the resulting inhibition zone diameters around the disks were measured and interpreted using the Clinical Laboratory Standards Institute (CLSI) breakpoints values [56,57,58]. Multidrug-resistant isolates (resistant to ≥ three antibiotic classes) were selected and stored in 20% (*v/v*) glycerol at −80 °C for future analyses.

### 4.6. Antibiotic Resistance Risk Assessment of Heterotrophic Bacterial Isolates

This was determined by the use of the Multiple Antibiotic Resistance Index (MAR-index), which was computed as (a/b), where “a” is the number of antibiotics to which the isolate was resistant, and “b” is the number of antibiotics to which the isolate was exposed. An isolate from an area of high antibiotic usage will demonstrate an MAR index of >0.2, while one with a value of <0.2 is from a source of lower antibiotic use.

### 4.7. Production of Extracellular Enzymes

Heterotrophic bacteria produced many extracellular enzymes, many of which are important in disease virulence. The MDR isolates were subjected to assays designated to determine their abilities to produce extracellular enzymes including hemolysin, proteinase, DNase, and lipase using standard methods.

Hemolysin production was determined by sub-culturing single colonies of pure isolates on 5% (*w/v*) sheep blood agar and incubating plates aerobically at 37 °C for 24 h. The presence of clear zones around the bacterial colonies indicated beta (β)-hemolysis, and a greenish coloration represented alpha (α)-hemolysis, while the absence of hemolytic patterns around colonies was interpreted as gamma (γ)-hemolysis.

Isolates were also screened for their potential to produce proteinases, which are enzymes that facilitate the catabolism of peptide bonds on long protein chains that bind amino acids. Single pure colonies of the test isolates were spot-inoculated on Brain Heart Infusion agar (Biolab, Modderfontein, South Africa) (pH 7.4 ± 0.2) supplemented with 3% (*w/v*) skimmed milk (Oxoid, Hampshire, UK). Plates were incubated aerobically at 37 °C for 24 h, and isolates that produced clear zones around the colonies were recorded as positive for proteinases.

For the potential to produce DNase, a single pure colony of each test isolate was sub-cultured on DNase agar (pH 7.3 ± 0.2) (Fluka Analytical, St. Gallen, Switzerland) supplemented with 0.01% toluidine blue O solution (Sigma Aldrich, US). Plates were incubated aerobically at 37 °C for 24 h and then flooded with a 0.1% (*v/v*) 1M HCl (Merck, US). Isolates that produced a zone of clearing or a pink halo around the bacterial colony indicated a positive reaction for the DNase enzyme.

Lipase is an enzyme that facilitates the catabolism of triacylglycerol into simpler compounds that include monoacylglycerols, diacylglycerols, free fatty acids, and glycerol. Single pure colonies of the test isolates were sub-cultured on Tryptone soy agar (Merck, Modderfontein, South Africa) supplemented with 1% (*v/v*) Tween 80 (Sigma Aldrich, St. Louis, MO, USA), which served as the substrate for the lipase enzyme. Plates were incubated aerobically at 37 °C for 24 h. The production of a turbid halo around bacteria colonies was indicative of a positive reaction for lipase enzyme.

### 4.8. Molecular Characterisation of Isolates

#### 4.8.1. Extraction of Chromosomal DNA

Genomic DNA was extracted from all presumptive multidrug-resistant (MDR) isolates using the Genomic DNA^TM^ Tissue Miniprep Kit (Zymo Research, Irvine, CA, USA), following the manufacturer’s instructions and quantified using a Nanodrop Lite spectrophotometer (Model 1558, Thermo Scientific, Waltham, MA, USA).

#### 4.8.2. Detection of Antibiotic Resistance Genes

The presence of antibiotic resistance genes was detected by PCR using specific primer sequences (Table 4). Each PCR reaction mixture comprised 25 μL reaction volumes consisting of 12.5 µL of a 2X DreamTag Green Master Mix (0.4 mM dATP, 0.4 mM dCTP 0.4 mM dGTP and 0.4 mM dTTP, 4 Mm MgCl_2_ and loading buffer), 0.25 µL of each oligonucleotide primer, 1 µL of template DNA and 11 µL of nuclease-free water. For multiplex PCR assays, appropriate volumes of nuclease-free water were added. A C1000 Touch^TM^ Thermal Cycler (Bio-Rad, Kidlington, UK) was used for all PCR amplifications, and amplicons were separated by electrophoresis on a 1.3% (*w/v*) agarose gel in a 1 × TAE buffer [20 mM Acetic acid (Merck, US), 40 mM Tris (Sigma Aldrich, US) and 1 mM EDTA (Merck, Modderfontein, South Africa) at pH 8.0] run at 70 V, for 60 min. A 100 bp DNA molecular weight marker was included in each run. Gels were stained in ethidium bromide (0.001 µg/mL) and visualized in a ChemiDoc Imaging System (Bio-RAD ChemiDoc^TM^ MP Imaging System, Gene snap version 6.00.22, Kidlington, UK). The oligonucleotide primers and PCR cycling conditions used in the study are shown in Table 4.

#### 4.8.3. Identification of MDR Isolates by Bacterial 16S rRNA Gene Analysis

The bacterial 16S rRNA gene fragments were amplified by PCR using the universal 27F and 1492R bacterial primers [67]. The resulting PCR products were sequenced at the Department of Microbiology, Potchefstroom Campus, South Africa. The sequence data were subjected to a blast search using the Blast Search Tool (http://blast.ncbi.nlm.nih.gov/Blast.cg) to determine the identities of the isolates.

### 4.9. Statistical Analysis

The means of triplicate measurements of the physicochemical and bacteriological water quality parameters were determined using Microsoft Excel. Antibiotic resistance data were subjected to analysis using SPSS v.26, and a Chi-square test was used to determine if there were any significant differences between the proportions of antibiotic-resistant isolates obtained before and after treatment for each study site. Statistical significance was set at *p* < 0.05.

## 5. Conclusions

In conclusion, the current study revealed good water quality in the Spring and Autumn seasons in the two-drinking water treatment and distribution systems; however, there were low aesthetic properties during the Summer and Winter seasons probably as a result of drainage into water catchments during rainfalls. Antimicrobial-resistant heterotrophic bacteria and genes were evident in the entire course of the water distribution systems, which may serve as sources for the transmission of resistant determinants to susceptible bacteria, including human pathogens. Approximately half of the tested isolates originated from areas of high antibiotic use. Some of the bacteria strains identified are of clinical importance and may pose treatment failures in the host during antibiotic therapy. The prevalence of antibiotic-resistant isolates and genes, especially in the potable water, indicates a need for further research to track and identify the sources of potential environmental contaminants, which may guide the development of strategies in preventing the development and spread of antibiotic resistance.

## Figures and Tables

**Table 1:** Mean physicochemical parameters from sites A and B throughout the sampling period.

Season		Temperature °C		pH		EC (µS/cm)		TDS (mg/L)	
	Blue Drop Limit [34]	-	-	≥5 to ≤9.7		≤170		≤1200	
	Site	A	B	A	B	A	B	A	B
Winter	Raw	18.7	10.4	9.53	7.76	800	405	508	827
	Inlet	16.5	21.0	9.40	8.55	776	289	489	182
	Outlet	18.5	21.2	8.94	8.52	846	309	533	195
	D1	21.0	22.2	9.17	7.73	750	805	473	507
	D2	19.6	27.0	9.62	8.17	656	360	413	192.9
Spring	Raw	25.7	24.6	9.94	8.96	763	390	481	246
	Inlet	23.9	26.5	9.93	8.79	774	305	487	192
	Outlet	24.8	24.5	9.47	8.85	773	321	487	202
	D1	27.0	31.1	9.17	8.56	703	321	443	202
	D2	28.3	26.5	8.37	8.78	677	305	426	192.2
Summer	Raw	28.5	24.5	7.42	7.84	283	262	180.9	167.3
	Inlet	22.5	25.2	7.37	7.64	239	270	152.3	169.2
	Outlet	22.3	24.8	7.47	7.6	256	271	164.1	172.9
	D1	27.8	26.8	7.34	7.64	287	280	183.2	179.6
	D2	26.4	29.2	7.37	7.61	876	284	559	182.5
Autumn	Raw	23.5	17.6	8.86	9.56	375	366	765	747
	Inlet	20.9	20.0	8.58	8.65	374	357	764	717
	Outlet	21.1	21.0	8.28	8.43	398	355	811	727
	D1	22.4	24.6	9.03	8.88	400	370	816	760
	D2	21.8	25.6	8.66	8.62	387	338	789	690

A—raw-untreated dam water, B—raw-untreated surface water reservoir, Inlet—abstracted water from catchment prior treatment, Outlet—water after treatment, D1: random household tap water from treatment plant distribution system, D2: second random household tap water from treatment plant distribution system.

**Table 2:** Average number of indicator bacteria obtained during the sampling period.

Site	Source	Total Coliform (cfu/100 mL) Blue Drop Limit, <10 [34]				Fecal Coliform (cfu/100 mL) Blue Drop Limit, 0 [34]				*E. coli* (cfu/100 mL) Blue Drop Limit, 0 [34]			
		Winter	Spring	Summer	Autumn	Winter	Spring	Summer	Autumn	Winter	Spring	Summer	Autumn
**A**	*Raw*	5	17	5	1	2	7	2	3	-	24	-	17
	*Inlet*	3	1	3	-	4	1	4	-	2	5	2	7
	*Outlet*	-	-	-	-	5	-	5	-	-	-	-	-
	*D1*	4	-	4	-	3	-	3	-	-	-	-	-
	*D2*	-	1	-	-	-	1	-	-	-	-	-	-
**B**	*Raw*	1	52	1	21	7	12	7	1	1	62	1	5
	*Inlet*	5	-	5	3	7	6	6	5	2	-	2	1
	*Outlet*	-	-	-	-	1	-	1	-	-	-	-	-
	*D1*	21	-	21	-	9	-	9	-	-	-	-	-
	*D2*	2	-	-	-	-	-	-	-	-	-	-	-

A—raw-untreated dam water; B—raw-untreated surface water reservoir; Inlet—abstracted water from catchment before treatment; Outlet—water after treatment; D1: random household tap water from treatment plant distribution system; D2: second random household tap water from treatment plant distribution system.

**Table 3:** Number and percentage of heterotrophic bacterial isolates resistant to the antibiotics.

Antibiotics	Site A		Site B		Total
	Before Treatment NT = 40 (28G+, 12G-)	After Treatment NT = 62 (41G+, 21G-)	Before Treatment NT = 37 (27G+, 10G-)	After Treatment NT = 64 (43G+, 21G-)	NT = 203 (139G+, 64G-)
AP *	15 (53.6)	25 (61.0)	25 (92.6)	24 (55.8)	89 (64)
PG *	12 (42.9)	24 (58.5)	19 (70.4)	17 (39.5)	72 (51.8)
VA *	3 (10.7)	7 (17.1)	10 (37.0)	9 (20.9)	29 (20.9)
E	1 (2.5)	3 (4.8)	2 (5.4)	2 (3.1)	8 (3.9)
CHL	6 (15)	11 (17.7)	8 (21.6)	10 (15.6)	35 (17.2)
KF	0 (0.0)	1 (1.6)	4 (10.8)	7 (10.9)	12 (5.9)
S	5 (12.5)	11(11.7)	4 (10.8)	11 (17.2)	31(15.3)
K	5 (12.5)	19 (30.6)	10 (27.0)	11 (17.2)	45 (22.2)
NE	1 (2.5)	3 (4.8)	3 (8.1)	8 (12.5)	15 (7.4)
OT	4 (10)	10 (16.1)	15 (40.5)	11 (17.2)	40 (19.7)
CIP	1 (2.5)	1 (1.6)	1 (2.7)	1 (1.6)	4 (2.0)
TM	17 (42.5)	31 (50)	27 (73.0)	30 (46.9)	105 (51.7)

AP—Ampicillin; PG—Penicillin G; VA—Vancomycin; E—Erythromycin; CHL—Chloramphenicol; KF—Cephalothin; S—Streptomycin; K—Kanamycin; NE—Neomycin; OT—Oxy-tetracycline, CIP—Ciprofloxacin, TM—Trimethoprim; G+—Gram-positive isolates, G-—Gram-negative isolates, *—tested only for Gram-positive bacteria. “Before treatment”—isolates recovered from the dam or water reservoir and inlet samples to each treatment plant; “After treatment”—isolates obtained following treatment (outlet) and the distribution systems (potable water).

**Table 4:** Oligonucleotide primers used in the study.

Target Gene	Primer Sequence (5′-3′)	Amplicon Size (bp)	Cycling Conditions	Reference
*erm* *(B)*	F: GATACCGTTTACGAAATTGG	364	58 °C for 1 min, 72 °C for 1 min, and a final elongation of 72 °C for 1 min.	[59]
	R: GAATCGAGACTTGAG TGTGC			
*sul1*	F: CCGTTGGCCTTCCTGTAAAG	67	94 °C for 5 min, 30 cycles of 94 °C 30 s, 58 °C for 1 min, 72 °C for 1 min, and a final elongation of 72 °C for 10 min.	
	R: TTG CCGATCGCGTGAAGT			
*tet(A)*	F: GCTACATCCTGCTTGCCTTC	210	95 °C for 1 min, 40 cycles of 95 °C 15 s, 62 °C for 1 min, 72 °C for 1 min, and a final elongation of 72 °C for 10 min.	[60]
	R: CATAGATCGCCGTGAAGAGG			
*tet(W)*	F: GAGAGCCTGCTATATGCCAGC	168		
	R: GGGCGTATCCAC AATGTTAAC			
*tet(X)*	F: AGCCTTACCAATGGGTGTAAA	278		
	R: TTCTTACCTTGGACATCCCG			
*mec(A)*	F: ATGCGCTATAGATTGAAAGGAT	163	95 °C for 1 min, 40 cycles of 95 °C for 15 s, 60 °C for 1 min for 1 min, 72 °C for 1 min, and a final elongation of 72 °C for 10 min.	
	R: TACGCGATATCTAACTTTCCTA			
*ampC*	F: TTCTATCAAMACTGGCARCC	1048	95 °C for 1 min; 35 cycles of 94 °C 30 s, 49 °C for 30 s, 72 °C for 1 min and a final elongation of 72 °C for 10 min.	[61]
	R: CCYTTTTATGTACCCAYGA			
*strA*	F: CTTGGTGATAACGGCAATTC	548	94 °C for 1 min, 30 cycles of 94 °C for 45 s, 58 °C for 45 s, 72 °C for 45 s and a final elongation of 72 °C for 7 min	[47]
	R: CCAATCGCAGATAGAAGGC			
*strB*	F: ATCGTCAAGGGATTGAAACC	509		
	R: GGATCGTAGAACATATTGGC			
*aadA*	F: GTGGATGGCGGCCTGAAGCC	525	95 °C for 4 min, 30 cycles of 94 °C for 45 s, 60 °C for 45 s, 72 °C for 45 s, and a final elongation of 72 °C for 10 min.	[62]
	R: AATGCCCAGTCGGCAGCG			
*cmlA*	F: CCGCCACGGTGTTGTTGTTATC	698	95 °C for 2 min, 30 cycles of 94 °C for 1 s, 40 °C for 1 s, 72 °C for 15 s, and a final elongation of 72 °C for 10 min.	[63]
	R: CACCTTGCCTGCCCATCATTAG			
*vanA*	F: GGGAAAACGACAATTGC	732	94 °C for 3 min, 32 cycles of 94 °C for 30 s, 60 °C for 45 s, 72 °C for 2 min, and a final elongation of 72 °C for 10 min.	[64]
	R: GTACAATGCGGCCGTTA			
*dfrB1, dfrB2*	F: CAAAGTAGCGATGAAGCCA	205	95 °C 10 min, 30 cycles of 95 °C for 15 s, 50 for 1 min, 72 °C for 1 min, and a final elongation of 72 °C for 10 min.	[65]
	R: CAGGATAAATTTGCACTGAGC			
*bla*_CTX-M_	F: ATGTGCAGYACCAGTAA	536	94 °C for 5 min, 40 cycles of 94 °C for 1 min, 57 °C for 1 min, 72 °C for 1 min, and a final elongation of 72 °C for 10 min.	[66]
	R: CCGCTGCCGGTYTTATC			
Bacterial 16S rRNA	F: AGAGTTTGATCATGGCTCAG	1420	94 °C, 3 min. 25 times (94°C, 1 min; 55°C, 1 min) 72 °C, 2 min.	[67]
	R: GGTACCTTGTTACGACTT			

## Supplementary Materials

The following are available online at https://www.mdpi.com/2079-6382/9/11/745/s1, Figure S1: Representative agarose gel image of the *strA* gene products. Lane M: 100bp DNA ladder; Lanes 1–11 and 13–14: *strA* positive gene fragments and Lane 12: negative control, Figure S2: Representative agarose gel of *strB* gene fragments amplified from isolates. Lane M: 100bp DNA ladder; Lanes 1–5, 7–9: *strB* positive fragments, and Lane 10: negative control, Figure S3: An agarose gel image of the *dfrB1* and *dfrB2* gene fragments amplified from isolates. Lane M:100 base pairs DNA ladder; Lanes 1–13: *dfrB1* and *dfrB2* positive gene fragments; Lane 14: negative control, Figure S4: An image depicting a 1.3% (*w/v*) agarose gel of the *aadA* gene fragments amplified from MDR isolates. Lane M: 100bp DNA ladder; Lanes 1–5: *aadA* gene fragments of representative isolates and Lane 6: negative control.
