# Supplementary material for: Occurrence of Antibiotic-Resistant Bacteria and Genes in Two Drinking Water Treatment and Distribution Systems in the North-West Province of South Africa
Source: Antibiotics (Basel). 2020 Oct 28;9(11):745. doi: 10.3390/antibiotics9110745 (PMC7692212; doi:10.3390/antibiotics9110745)
Supplement: Supplementary file 1 [file antibiotics-09-00745-s001.pdf]

# Supplementary Materials

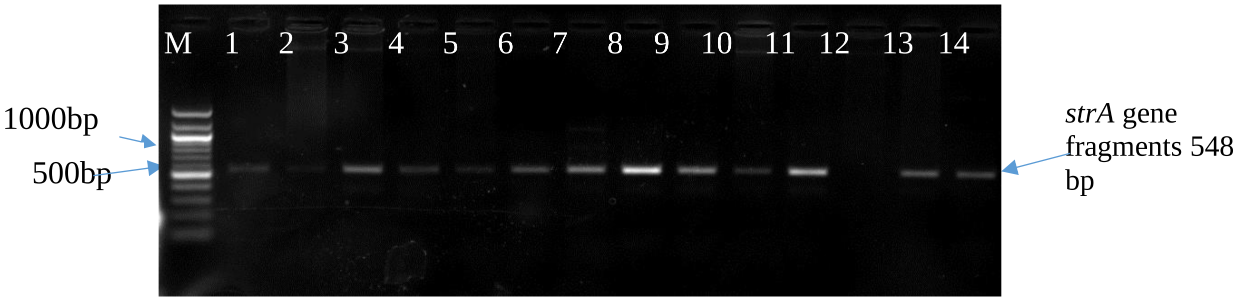


**Figure S1:** Representative agarose gel image of the *strA* gene products. Lane M: 100bp DNA ladder; Lanes 1-11 and 13-14: *strA* positive gene fragments and Lane 12: negative control.

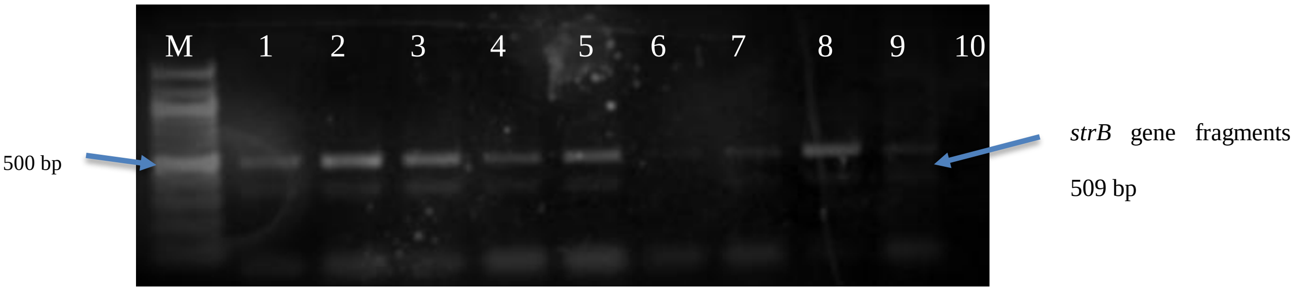


**Figure S2:** Representative agarose gel of *strB* gene fragments amplified from isolates. Lane M: 100bp DNA ladder; Lanes 1-5, 7-9: *strB* positive fragments, and Lane 10: negative control.

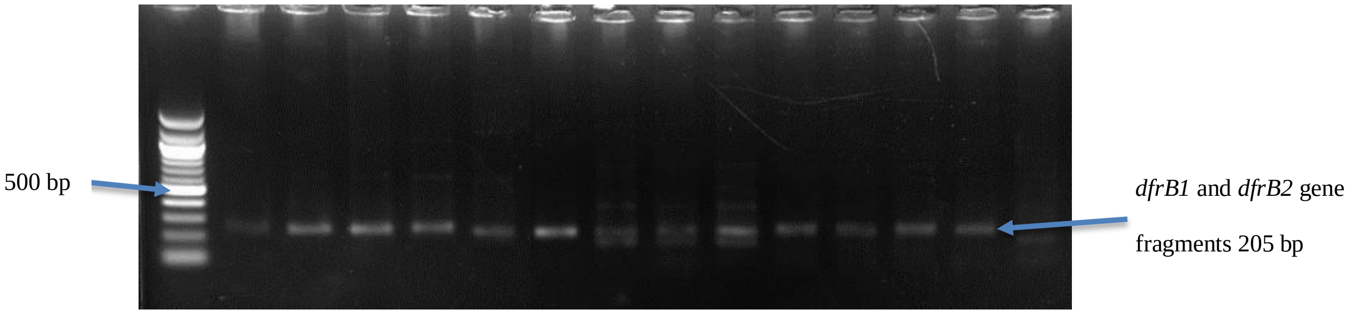


**Figure S3:** An agarose gel image of the *dfrB1* and *dfrB2* gene fragments amplified from isolates. Lane M:100 base pairs DNA ladder; Lanes 1-13: *dfrB1* and *dfrB2* positive gene fragments; Lane 14: negative control.

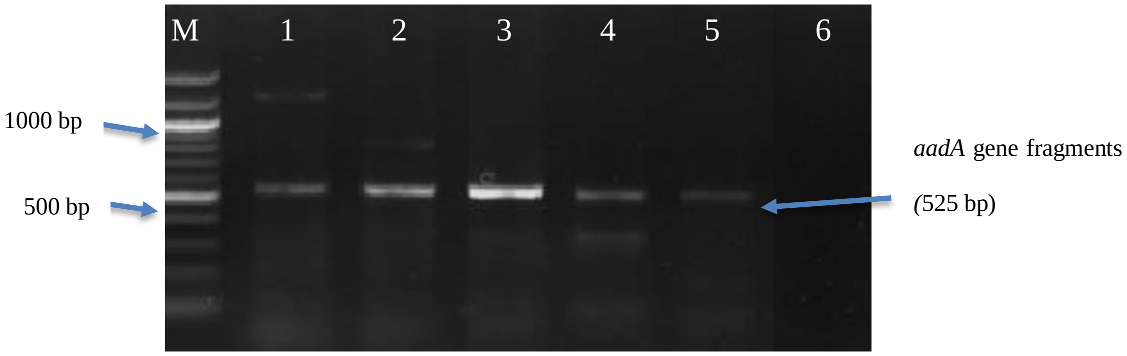


**Figure S4:** An image depicting a 1.3% (w/v) agarose gel of the *aadA* gene fragments amplified from MDR isolates. Lane M: 100bp DNA ladder; Lanes 1-5: *aadA* gene fragments of representative isolates and Lane 6: negative control.
